# A C-type lectin with an immunoglobulin-like domain promotes phagocytosis of hemocytes in crayfish *Procambarus clarkii*

**DOI:** 10.1038/srep29924

**Published:** 2016-07-14

**Authors:** Xiao-Wen Zhang, Yue Wang, Xian-Wei Wang, Lei Wang, Yi Mu, Jin-Xing Wang

**Affiliations:** 1 College of Life Science, Henan Normal University, Xinxiang, Henan, 453007, China; 2 Shandong Provincial Key Laboratory of Animal Cells and Developmental Biology, School of life science, Shandong University, Jinan, Shandong, 250100, China

## Abstract

C-type lectins are important immune molecules that participate in host defense response. The present work reports a novel C-type lectin (*PcLec3*) from the red swamp crayfish *Procambarus clarkii.* Sequence analysis found that *PcLec3* encodes a polypeptide with252 amino acid residues, which contains an immunoglobulin-like domain (IG) and a C-type lectin domain (CTLD) arranged in tandem. Tissue distribution analysis indicated that *PcLec3* is enriched expressed in hemocytes and hepatopancreas cells, in which *PcLec3* was up-regulated following bacterial challenge by *Vibrio anguillarum*. Function analysis using recombinant full-length PcLec3, IG, and CTLD proteins revealed that these recombinant proteins had the capacity to bind carbohydrates and bacteria, while IG determined the cell binding activity. However, only full-length PcLec3 promotes the phagocytic activity of hemocytes and subsequent clearance of invasive bacteria. Taken together, these results manifest that PcLec3 acts as a hemocyte adhesion molecule to promote hemocyte phagocytosis against invasive *V. anguillarum*.

Animals can initiate host defense against invading pathogens. Adaptive immunity, which has long been thought to be restricted to vertebrates, is mediated by the immunoglobulins (Igs) and T-cell receptors. Proteins in the immunoglobulin superfamily (IgSF) possess at least one Ig domain, and are involved in the recognition, binding or adhesion processes of cells^1^. Generally, IgSF proteins are encountered more frequently in vertebrates than invertebrates. However, several invertebrate genes possessing Ig-like domains (IGs) have been reported to be involved in host defense. The Down Syndrome cell adhesion molecules (Dscams) in several arthropods, including crustaceans, implied their alternative adaptive immunity^2^,^3^,^4^,^5^. These molecules are capable of alternative splicing to form mRNA variants^6^. In the snail *Biomphalaria glabrata*, circulating hemocytes secrete fibrinogen-related proteins (FREPs) with IgSF and fibrinogen domains in response to trematode infections^7^,^8^. The hemolin from the hemocyte of giant silkmoth *Hyalophora cecropia* is likewise a member of IgSF, which binds to bacterial surfaces using its different cell adhesion properties^9^.

Aside from the IgSF proteins, there are several C-type lectins (CTLs) containing Ig-like domains. CTLs are generally accepted as pattern recognition receptors (PRRs), which identify the pathogen-associated molecular patterns (PMAPs) exposed on pathogen surface and participate in various immune defense mechanisms^10^. These receptors usually contain the C-type lectin domain (CTLD) and are divided into different groups in vertebrates according to the presence of other motifs^11^. The function of CTLs in crustacean immune systems, especially in shrimps, has been well reviewed by Wang *et al*.^12^. Moreover, CTLs are considered having great potential to generate high immune specificity, due to their abundance and diversity in most metazoan genomes^13^. As one member of the CTL superfamily, IG-containing CTLs have been reported in several studies. Lecticans, which are lectins with an IG and one or more epidermal growth factor domains, are involved in the development and organization of the extracellular matrix by binding to hyaluronan or chondroitin sulfate^14^,^15^. Another CTL with several IG and fibronectin type 3 domains was identified in *Drosophila*^16^. Other than these, few groups have studied CTLs that contain only IG domains, and no reports have been published on their functional analysis. The *BSCLT* gene that was cloned from *Botryllus schlosseri* has a CTLD and an IG at its N- and C-terminals, respectively^17^. In contrast, the BgSel gene and C-type lectin-related proteins (CREPs) from *B. glabrata* have an N-terminal IG and C-terminal CTLD^18^,^19^.

Considering IgSF and CTLs are both closely related to immunity, it is reasonable to speculate IgSF-containing CTLs have specific roles in innate immune defense. The functions of each domain and their interaction should be explored. This study reports an IG-containing CTL, which was designated as *PcLec3*, from the red swamp crayfish *Procambarus clarkii*. The mRNA and protein levels of PcLec3 were up-regulated upon bacterial stimulation by *Vibrio anguillarum*. Recombinant full-length PcLec3 (rPcLec3) and recombinant forms of each domain (rIG or rCTLD) were used to investigate their specific activities. Bacterial clearance and RNA interference experiments were conducted to test the role of PcLec3 in the anti-bacterial defense of crayfish. To the best of our knowledge, this is the first report to analyze the specific functions of an IG-containing CTL in crustacean.

## Results

### PcLec3 is an IG containing C-type lectin

**Figure 1:**
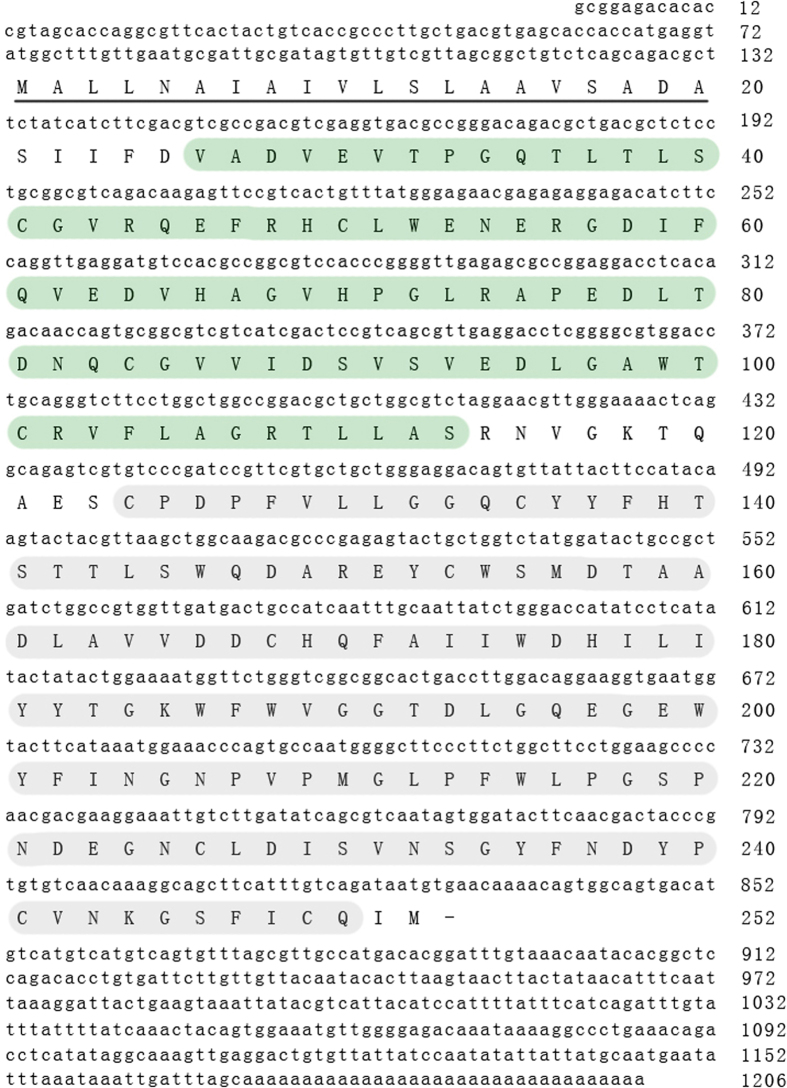
The cDNA and deduced amino acid sequences of *PcLec3* from *P. clarkii.* The amino acid residues (26–113) of IG domain is identified by the green background, and the gray background shows the CTLD amino acid residues (124–250).

Full-length cDNA of *PcLec3* was obtained by random sequencing of the cDNA library from crayfish (*P. clarkii*). The 758 bp open reading frame of *PcLec3* encodes a protein of 252 amino acids (GenBank accession no.JX844151) with an IG, a CTLD, and a signal peptide domain, as predicted by the SMART program (Fig. 1).

The PcLec3 IG that was predicted by SMART resembles most an Ig kappa light chain variable region. The PcLec3 IG contains the two cysteine residues that form the IG typical intrachain disulfide bridge and the tryptophan residue packed against the disulfide (Supplementary Fig. S1A), which is a predominant feature of most Ig domains. This IG shares similarity with Ig homologs from *Monodelphis domestica, Trichosurus vulpecula*, and *Macropus eugenii*. Interestingly, Blast search also found the IG of PcLec3 also shares identity with a putative cell adhesion protein (CA) from *Ixodes scapularis*. The multiple sequence alignments of amino acid sequences from these five proteins showed that 12 conserved amino acids were found in IG domain (Supplementary Fig. S1A). Several CTLs from the BLAST results were chosen to construct a phylogenetic tree with full-length PcLec3 (Supplementary Fig. S1B). PcLec3 was clustered into the invertebrate CTLs, although they share low identity, possibly due to its unique IG-containing structure.

### PcLec3 was up-regulated after the V. anguillarum challenge

**Figure 2:**
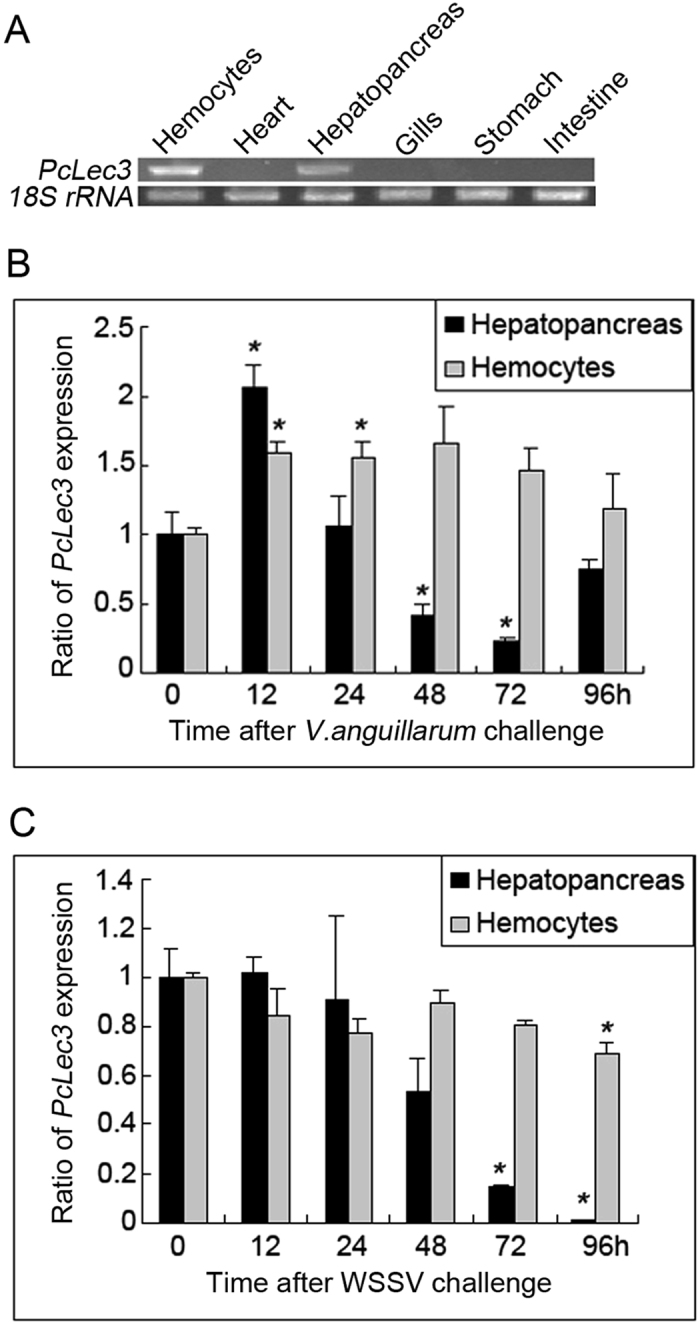
Expression profiles of *PcLec3* transcripts. (**A**) Semi-quantitative PCR detected *PcLec3* mRNA in the hemocytes and hepatopancreas of normal crayfish. Time course expression of *PcLec3* of crayfish challenged with (**B**) *V. anguillarum* or (**C**) WSSV analyzed by qRT-PCR. The asterisks (*) indicate significant differences (*P* < 0.05) between the challenged and unchallenged crayfish. Error bars represent ± SD of the three independent PCR amplifications and quantifications.

Tissue distribution analysis revealed that *PcLec3* was detected in the hemocytes and the hepatopancreas of normal crayfish (Fig. 2A). Furthermore, the time course expression profile of *PcLec3* was analyzed in crayfish challenged with *V. anguillarum* (Fig. 2B) or white spot syndrome virus (WSSV) (Fig. 2C). In hepatopancreas, *PcLec3* was significantly up-regulated at 12 h post injection (PI) with bacteria challenge and then recovered to the basal level at 24 h PI. The expression of PcLec3 maintained initially at a normal level and then gradually declined at 24 h PI with WSSV challenge. In hemocytes, PcLec3 was slightly up-regulated after the *V. anguillarum* challenge, whereas no obvious change was observed after the WSSV challenge.

### PcLec3 protein was secreted to cell-free hemolymph after bacterial challenge

**Figure 3:**
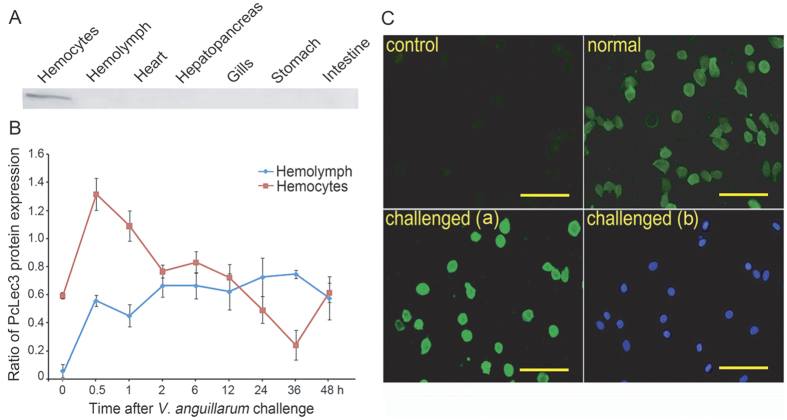
Expression profiles of PcLec3 protein. (**A**) Western blot analysis of PcLec3 protein in various tissues of normal crayfish. (**B**) Time course expression of PcLec3 in hemocytes and hemolymph. (**C**) Hemocyte immunohistochemistry. The panel shows hemocytes (normal or challenged by bacteria) incubated with polyclonal rabbit antiserum to PcLec3 and hemocytes incubated with normal rabbit serum as a control. The green color of the normal and challenged crayfish (a) shows the FITC-conjugated goat anti-rabbit IgG, and the blue color in challenged crayfish (b) shows the same visual field with challenged crayfish (a), indicating the location of the nucleus. Bar = 20 μm.

The antiserum against recombinant PcLec3 was used to detect the protein expression profiles of PcLec3. Western blot analysis results showed the PcLec3 protein was originally detected only in the hemocytes of unchallenged crayfish and in the circulatory hemolymph 0.5 h after the bacterial injection (Fig. 3A). In the hemocytes, the *V. anguillarum* challenge lead to dramatic increase in the PcLec3 protein level and then this high-level expression began to gradually return to basal level 2 h after bacterial stimulation. Correspondingly, in the hemolymph *V. anguillarum* challenge also result in high-level and stable expression of PcLec3 protein for at least two days (Fig. 3B). This phenomenon was attributed to the secretion of PcLec3 from the hemocytes into the cell-free hemolymph. Further immunocytochemistry analysis revealed that PcLec3 signal in bacterial-challenged crayfish was significantly higher than that in unchallenged crayfish (Fig. 3C), consistent with the results in Western blot analysis (Fig. 3B).

### Recombinant PcLec3 and its two domains can bind to microorganisms and sugars

For further functional analyses, the mature PcLec3 and its individual domains (IG and CTLD) were subjected to recombinant expression in *E. coli* BL21 (DE3). All recombinants were fused with a His-tag at the N terminal. The size and purity of the recombinant proteins were confirmed with SDS-PAGE (Supplementary Fig. S2). The antiserum against recombinant PcLec3 was prepared using the recombinant proteins.

**Figure 4:**
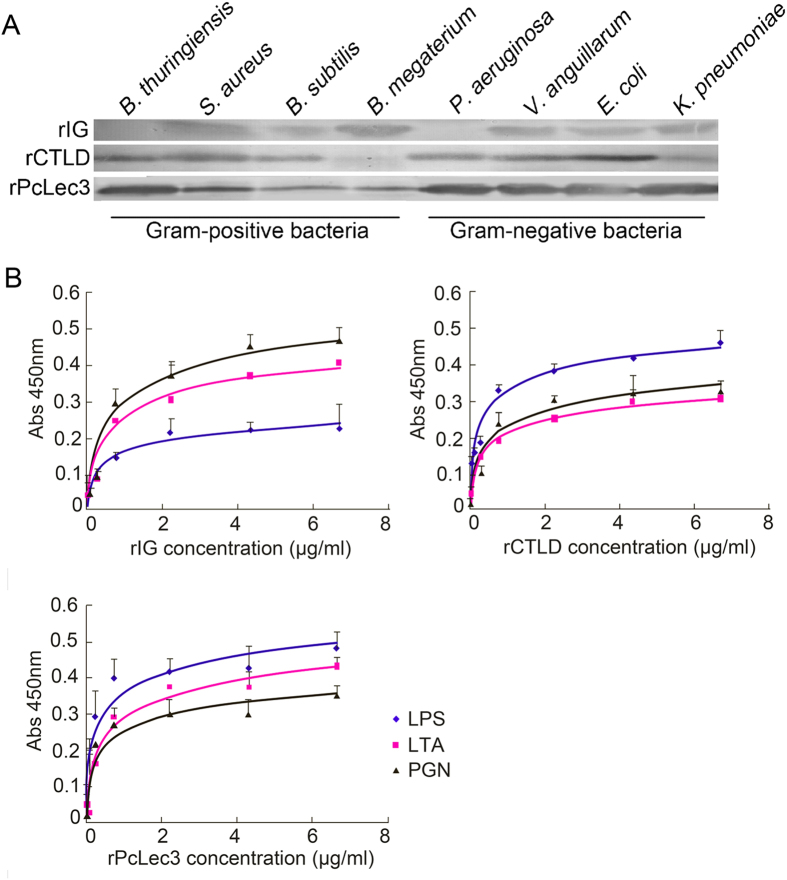
(**A**) PcLec3, IG, and CTLD all had bacterial binding ability. The recombinant proteins were detected by antiserum against rPcLec3. (**B**) Pclec3 and its two domains had sugar binding ability. Various amounts of rIG, rCTLD, or rPcLec3 were incubated with polysaccharides (PGN, LPS, or LTA) in the microtiter plates, and antiserum against rPcLec3 was used to detect the binding. Data shown are the mean ± SEM derived from the three repeats.

Using recombinant proteins and the antiserum, the microbial binding activity of rPcLec3 and its two domains were tested (Fig. 4A). Results manifested that PcLec3 did bind to all the microorganisms tested, while IG and CTLD did not bind to the G^+^ bacteria *P. aeruginosa* and *B. megaterium*, respectively. The sugar-binding ability was test by ELISA, and results indicated that recombinant proteins bound to polysaccharides, exhibiting a dose-dependent pattern. Each of the recombinant proteins can bind to the three kinds of polysaccharides (PGN, LPS, and LTA) with different binding affinities. IG had the lowest LPS binding affinity, compared with that of CTLD and PcLec3 (Fig. 4B).

### IG can bind to hemocytes

**Figure 5:**
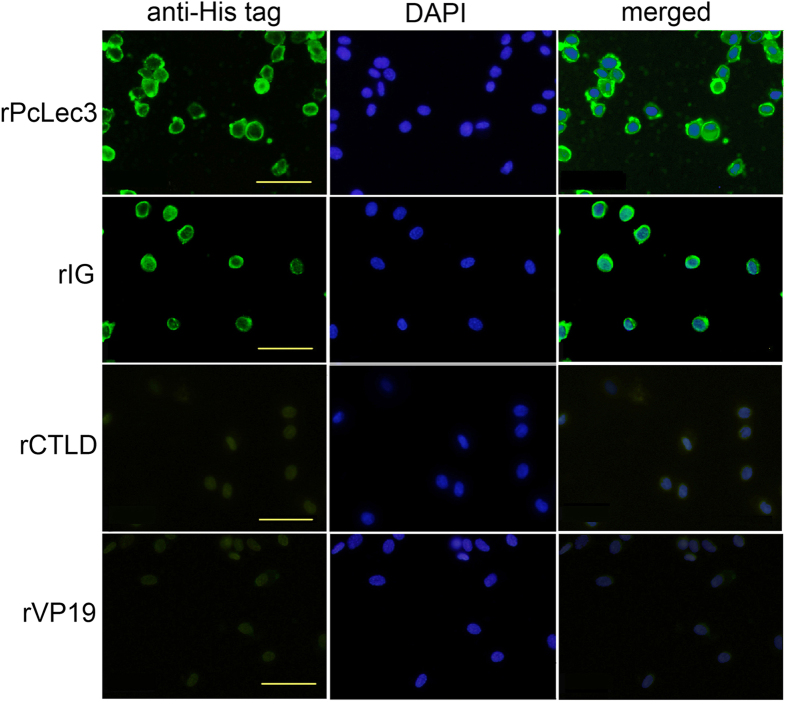
Hemocyte binding and cell adhesion properties of PcLec3. Recombinant proteins (rPcLec3, rIG, and rCTLD) were injected into the crayfish, and the hemocytes were isolated for immunohistochemistry assay to detect the binding property. The left panel shows the binding signal detected by the anti-His tag antibody (green), the middle panel indicates the corresponding nucleus location (blue), and the right panel is the merge of the two panels. Bar = 20 μm.

The cell binding ability of PcLec3 and its two domains were tested since many IgSF members possess cell recognition capability. The anti-His-tag antibody was used to detect if recombinant proteins can bind to cell surface of hemocytes, while the FITC-conjugated goat anti-mouse IgG was used as the secondary antibody, which can be visualized under fluorescent microscope. Obvious green signals on the edge of the hemocytes from the crayfish injected with rPcLec3 or rIG were observed (Fig. 5); however, no signal was observed in the hemocytes from rCTLD injected group. These results indicated that the IG domain, but not the CTLD domain of PcLec3, had cell-binding ability. We also found hemocytes adhered to each other in the PcLec3-injected group (Fig. 5), clearly different from the other groups, suggesting only the full-length PcLec3 had cell adhesion activity.

### Only full-length PcLec3 promoted the hemocyte phagocytosis

**Figure 6:**
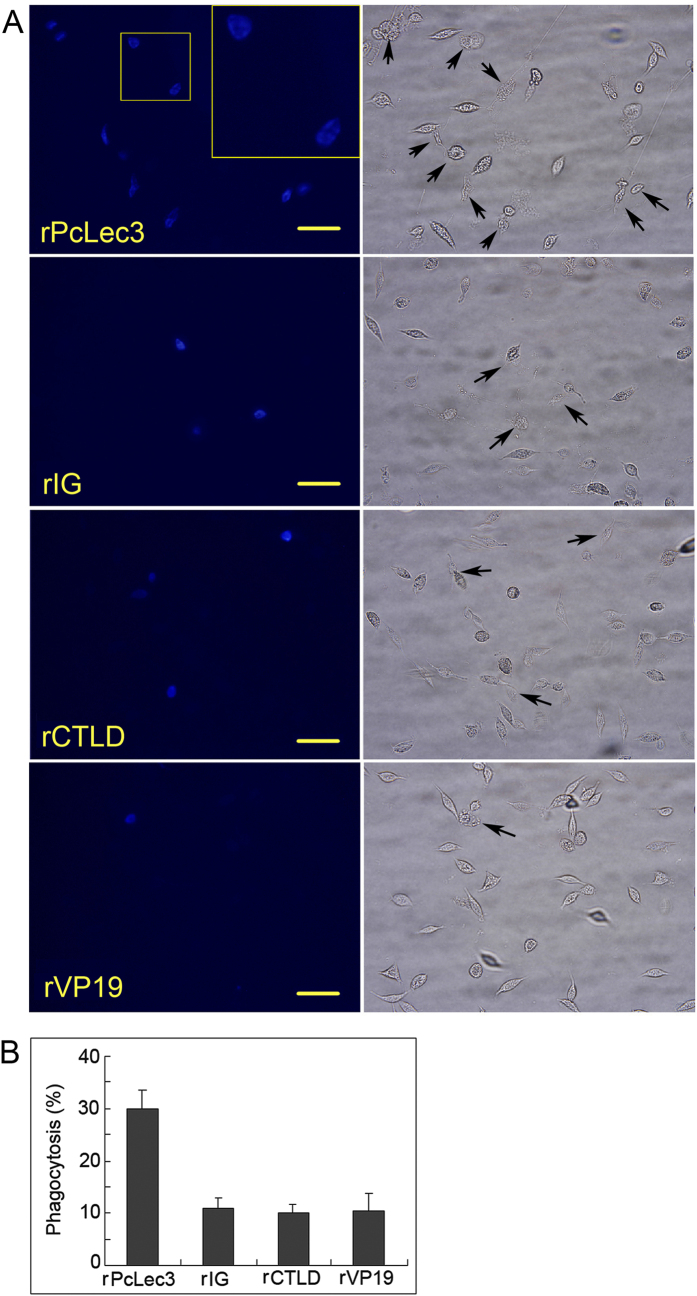
Hemocytic phagocytosis activity was promoted by the PcLec3 coating. (**A**) Left panel shows hemocytes undergoing phagocytosis, appearing as bright blue color (the DAPI dyed engulfed bacteria). (**B**) Right panel shows the same observations under white light, and the black arrows indicate the cells involved in phagocytosis. The phagocytic percentage was calculated as follows: (no. of cells ingesting bacteria/no. of cells observed) × 100%. The assays were conducted in triplicate.

The effect of PcLec3 on hemocyte phagocytosis was investigated since PcLec3 has binding capacity with bacteria and hemocytes. Phagocytosis signals were indicated with blue fluorescence in hemocytes, resulted from the DAPI stained bacteria (Fig. 6A). Pre-experiment found that phagocytosis signals were observed at 30 min PI. Therefore, the time point at 30 min PI was chosen to calculate the phagocytic percentage of each group (Fig. 6A,B). About 30% of the hemocytes participated in the bacterial binding with PcLec3, whereas only about 10% of the hemocytes had ingested the bacteria that displayed the other recombinant proteins (IG, CTLD or V19). These results demonstrated that only the full-length PcLec3 promoted hemocyte phagocytosis.

### CTLD assisted the bacterial clearance

**Figure 7:**
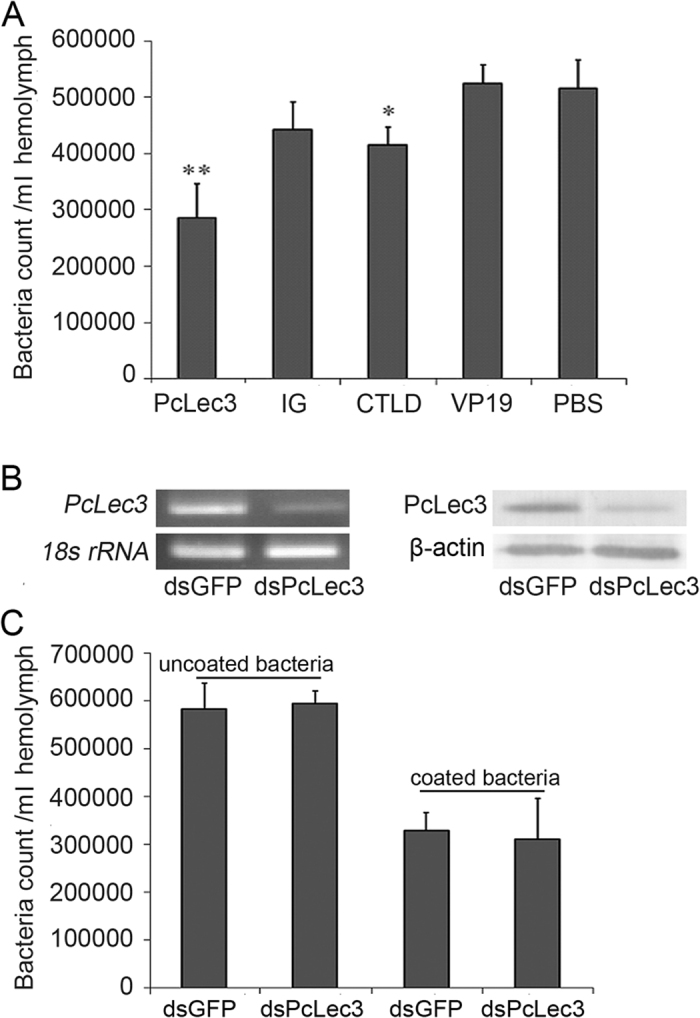
Roles of PcLec3 and its two domains in bacterial clearance. (**A**) Bacteria clearance experiment in different coatings of *V. anguillarum* with different proteins. Asterisks indicate the significant differences (*P < 0.05; **P < 0.01). (**B**) Efficiency of RNA interference as determined by RT-PCR and Western blot analysis. (**C**) Bacteria clearance experiment upon RNA interference with dsPcLec3 or dsGFP (control). Uncoated bacteria were not treated, and coated bacteria were coated with rPcLec3.

Given that PcLec3 promoted hemocyte phagocytosis, either the full-length proteins or its different domains were used to coat *V. anguillarum* to investigate whether these peptide sequences could facilitate the clearance of bacteria *in vivo*. Results manifested that coating with the full-length PcLec3 significantly promoted the clearance of *V. anguillarum* (Fig. 7A). Similar effects were observed using the CTLD coating. In contrast, the IG coating did not assist the bacterial clearance, thereby suggesting the bacteria-clearance facilitating function of PcLec3 is determined by CTLD. Further RNAi-mediated knockdown of PcLec3 manifested the successful knockdown of PcLec3 in both transcriptional and protein levels (Fig. 7B), but the clearance of the bacteria was not influenced (Fig. 7C), thereby suggesting that similar molecules maybe compensate for the loss of PcLec3. When the uncoated bacteria was substituted with the coated bacteria, the promoting effect was then rescued (Fig. 7C).

## Discussion

Igs in the immune system recognize and neutralize foreign targets such as bacteria and viruses in mammals^20^. The immunoglobulin domain is capable of facilitating molecular interactions and is an essential component of mammalian immunity. The immunoglobulin superfamily (IgSF) is a large group of molecules participating in the recognition, binding or adhesion processes of cells, and it is best known from the invertebrate immune system^1^. To date, several IgSF proteins have been reported in invertebrates, such as Dscam and FREPs^2^,^3^,^4^,^5^,^6^,^7^,^9^. Here, we report that PcLec3, a CTL from crayfish, is a member of the IgSF. PcLec3 contains an IG with a variable region of the light chain, which is not very highly conserved (Supplementary Fig. S1A).

PcLec3 belongs to the CTL family because of its CTLD (Fig. 1). CTLs are closely related to immunity because they can function as PRRs. Therefore, we hypothesized that PcLec3 participates in the innate immune defense of crayfish. The expression profiles of PcLec3 manifested that the PcLec3 transcripts were distributed in hemocytes and hepatopancreas (Fig. 2A), which are both important tissues responsible for immunity in crustaceans^21^. Upon the *V. anguillarum* challenge, the transcript level of PcLec3 was up-regulated (Fig. 2B). PcLec3 protein was detected only in hemocytes at normal condition (Fig. 3A). However, after the bacterial challenge, the PcLec3 signal increased in the hemocytes and even appeared in the hemolymph (Fig. 3B,C). These results revealed that the bacterial challenge induced PcLec3 expression and secretion, which suggest that PcLec3 is involved in anti-bacterial defense.

Furthermore, the mechanism of PcLec3 participation in anti-bacterial defense and the specific function of its two domains were investigated. Based on the literature, Ig binds to complementary molecules in mammals to mark the pathogens for phagocytosis or binds to pathogens and link them together to cause agglutination^22^, while CTLs possess the sugar-binding activity and the microorganism-binding ability to distinguish the pathogens^12^,^23^,^24^,^25^. Therefore, we performed bacterial and sugar binding assays. The results showed that both IG and CTLD can bind to most of the bacteria tested and to all the three polysaccharides (PGN, LPS, and LTA; Fig. 4). These results suggest that bacterial binding is one mechanism by which PcLec3 participates in anti-bacterial defense. Furthermore, PcLec3 had a higher affinity to the three carbohydrates and a wider bacterial binding ability than each of the domains (Fig. 4). This is reasonable because the binding ability of PcLec3 could be a combination of the abilities of the individual domains.

In addition to the pathogen-recognition ability, members of IgSF are commonly involved in either cellular or antigen recognition^26^. In the giant silkmoth *H. cecropia*, the IgSF member hemolin was reported to have cell adhesion properties^9^. In the present study, IG similarly bound to the hemocytes, but CTLD did not have this property (Fig. 5). Furthermore, we found that full length PcLec3 could cause hemocytes to adhere to each other, in the absence of bacteria (Fig. 5). Since IG itself is sufficient for cell binding but only full-length PcLec3 cause hemocytes adhere to each other, we speculate that CTLD of PcLec3 can oligomerize to bring PcLec3 molecules together, subsequently cause hemocytes adhesion since the IG of PcLec3 binds to hemocytes.

As the binding ability of PcLec3 links the bacteria and hemocytes together, it is reasonable to speculate that phagocytosis towards bacteria is enhanced. Results of the phagocytosis assay confirmed this hypothesis. PcLec3 enhanced the phagocytosis rate, whereas the individual IG or CTLD did not (Fig. 6). This result is similar to the studies of the immune cells in mammals, which have Fc receptors that recognize the pathogens marked by Ig and interact with IgA, IgG, and IgE to trigger further responses, including phagocytosis^27^. Further bacteria clearance experiment confirmed the function of PcLec3 in phagocytosis (Fig. 7A). However, PcLec3 knocked down by RNA interference using dsPcLec3 did not affect the clearance rate (Fig. 7B,C). This effect might be a result of the compensation by other molecules similarly promoting bacteria clearance, or the low amount of PcLec3 is sufficient to maintain the basal phagocytosis effect of hemocytes.

In summary, the present study revealed the CTLD of PcLec3 had bacterial binding ability, whereas the IG of PcLec3 possessed both bacteria and hemocyte binding abilities. The combination of these two domains causes the cell adhesion ability of the full-length PcLec3 and promotion of hemocyte phagocytosis of the invading bacteria.

## Materials and Methods

### Chemicals and microorganisms

Lipopolysaccharide (LPS; from the *Escherichia coli* serotype 055:B5), lipoteichoic acid (LTA; from *Staphylococcus aureus*), and peptidoglycan (PGN; from *Micrococcus luteus*) were purchased from Sigma (St. Louis, MO, USA). *Bacillus thuringiensis, Bacillus subtilis, Bacillus megaterium, Pseudomonas aeruginosa*, and *Klebsiella pneumoniae* were obtained from Shandong Agricultural University. The *V. anguillarum* strain was a gift from the Institute of Oceanology of the Chinese Academy of Sciences. Readily available *E. coli* and *S. aureus* cultures were maintained by our laboratory.

### Immune challenge and tissue collection in crayfish

*P. clarkii* individuals (12 g to 15 g each) were obtained from a market in Jinan, Shandong Province, China and cultured in tanks. All experiments carried on the crayfish, including sacrifice, were conducted according to the regulations of local and central government, which complied with the National Institute of Health Guide for the Care and Use of Laboratory Animals. The crayfish were injected with *V. anguillarum* in the abdominal segment (2 × 10^7^ cells per crayfish) for the immune challenge. The white spot syndrome virus (WSSV) immune challenge was performed according to a previously described method^28^. The hemolymph was collected from the ventral sinus using a 1 ml sterile syringe preloaded with 100 μl anticoagulant [0.14 M NaCl; 0.1 M glucose; 30 mM trisodium citrate; 26 mM citric acid; 10 mM EDTA (ethylenediaminetetraacetic acid), pH 4.6] as described by Jiravanichpaisal *et al*.^29^. Hemolymph collection was performed at 12, 24, 48, 72, and 96 h post-injection (PI). The collected hemolymph was centrifuged immediately at 800 × *g* for 5 min at 4 °C to isolate the hemocytes. Other tissues from the heart, hepatopancreas, gills, stomach, and intestines were likewise collected for RNA extraction.

### cDNA cloning

Total RNA was extracted from hepatopancreas and gill samples at 96 h post-WSSV challenge using the Unizol reagent (Biostar, Shanghai, China) by following the manufacturer’s protocol. PolyA Tract mRNA isolation system (Promega, USA) was used to extract the mRNA and then construct a cDNA library. For cDNA library construction, the Creator SMART cDNA Library Construction Kit (Clontech, USA) was used according to the manufacturer’s instructions. Double-stranded cDNA was ligated to the pDNR-LIB vector and transformed into *E. coli* DH5α competent cells. Individual colonies were randomly selected for plasmid extraction and sequencing. More than 5000 expressed sequence tags (ESTs) were obtained. The ESTs were analyzed using a basic local alignment search tool (BLAST) search (http://blast.ncbi.nlm.nih.gov/Blast.cgi). A CTL with an IG domain (designated as *PcLec3*) was selected for further studies.

### Sequence analysis

Deduced protein forecasting was performed using ExPASy (http://www.expasy.ch/), and the domains were predicted using the simple modular architecture research tool (SMART) (http://smart.embl-heidelberg.de/). The alignment of IG domains and phylogenetic tree construction of selected CTLs were achieved using the MEGA 4.0 program^30^.

### Quantitative real-time polymerase chain reaction (qRT-PCR)

The total RNA from a variety of tissues (hemocyte, heart, hepatopancreas, gill, stomach, and intestine) was extracted using the Unizol reagent (Biostar, China) according to the manufacturer’s protocol. Total RNA (5 μg) was then reverse transcribed, and synthesized first-strand cDNA was used as a template for qRT-PCR. The RealF and RealR primers (Table 1) were used to analyze the tissue distribution and expression patterns of *PcLec3* at different time points after the immune challenge. The 18 S rRNA gene, as the internal control, was amplified with the gene-specific primers 18SF and 18SR (Table 1). qRT-PCR was performed using a real-time thermal cycler (Bio-Rad, USA) following the protocol of the SYBR real-time PCR Premixture (Bioteke, China). The following qRT-PCR program was used: 95 °C for 3 min, followed by 40 cycles of 95 °C for 10 s and 68 °C for 30 s. The melt curves from 60 °C to 95 °C were then determined. The qRT-PCR data of *PcLec3* expression levels in response to bacterial and viral challenges were calculated by 2^−ΔΔ*C*^_T_ (ΔΔ*C*_T  _ = Δ*C*_TPcLec3_ − Δ*C*_T18S rRNA_). Obtained data were subjected to statistical analysis, Unpaired student’s *t*-test was employed to calculated the *P* value base on the normalized data, and *P* < 0.05 was considered statistically significant.

### Recombinant expression of PcLec3 and its two domains and antibody preparation

A DNA fragment encoding the mature PcLec3 and its two domains, IG and CTLD, was amplified using primers with restriction enzyme sites (i.e., ExF and ExR; IGEXF and IGEXR; CTLDEXF and CTLDEXR; Table 1). The cDNA fragments and pET30a(+) were digested with the corresponding restriction enzymes and then ligated together. Recombinant plasmids were used to transform *E. coli* BL21 (DE3) cells for recombinant protein expression. rPcLec3 and its individual domains (rIG and rCTLD) were expressed and purified as described previously^31^. Purified PcLec3 protein was used as an antigen for antiserum preparation in New Zealand rabbits according to the method described by Du *et al*.^31^. Briefly, 500 μg purified protein was emulsified in complete Freund’s adjuvant and then injected subcutaneously into the rabbit as the initial challenge; another 500 μg protein emulsified in incomplete Freund’s adjuvant was injected subcutaneously as the second challenge after 3 weeks; then 800 μg protein dissolved in PBS was injected into the muscle of the rabbit as the third challenge after 2 weeks; the last challenge was also injecting 800 μg protein into the muscle of the rabbit after 1 week. The rabbit blood was collected after 1 week and the serum was stored at −20 °C. The antibody titer was determined by double immunodiffusion method.

### Western blot analysis

Crayfish tissues (hemocyte, heart, hepatopancreas, gill, stomach, and intestine) were homogenized in the lysis buffer (50 mM Tris–HCl, pH 7.5; 150 mM NaCl; 1 mM PMSF, phenylmethylsulfonyl fluoride; 3 mM EDTA) and centrifuged at 12,000 × *g* for 10 min at 4 °C. The supernatant was collected and the Bradford method was used to determine the protein concentration^32^. Total proteins (100 μg) from each tissue were subjected to 12.5% sodium dodecyl sulfate polyacrylamide gel electrophoresis (SDS-PAGE) according to the Laemmli method^33^. The separated proteins on the gel were transferred onto a nitrocellulose membrane, which was blocked by incubating it in 3% non-fat dry milk in Tris-buffered saline (TBS; 10 mM Tris–HCl, pH 7.5; 150 mM NaCl) for 1 h. Then, the membrane was incubated with polyclonal rabbit antiserum against PcLec3 (1:100 dilution). Antibody binding was visualized through a colorimetric reaction catalyzed by the peroxidase-conjugated goat anti-rabbit IgG (1:10,000 dilution in TBS).

For the temporal expression of PcLec3 in crayfishes challenged with *V. anguillarum*, hemolymph (500 μl) was collected using a 1 ml sterile syringe containing 500 μl anticoagulant at 0.5, 1, 2, 6, 12, 24, 36, and 48 h PI. Hemolymph was centrifuged immediately at 800 × *g* for 5 min at 4 °C to isolate the hemocytes. Proteins of the hemocytes and the hemolymph without hemocytes were sampled and used for Western blot analysis.

### Microorganism binding assay

Gram-positive (G^+^) bacteria (*B. thuringiensis, S. aureus, B. subtilis*, and *B. megaterium*) and Gram-negative (G^−^) bacteria (*P. aeruginosa, V. anguillarum, E. coli*, and *K. pneumoniae*) were selected for the binding assay. The bacteria were cultured in lysogeny broth (LB; 1% tryptone, 1% NaCl, 0.5% yeast extract) overnight and collected by centrifugation at 6,000 × *g* for 5 min, washed with TBS, and then resuspended in TBS to an optical density at 600 nm (*OD*_600_) of 1.0. Purified recombinant PcLec3 (1 mg/ml; 50 μl) was added and incubated with 500 μl of the microbial suspension (2 × 10^7 ^cells/ml) with rotation at room temperature for 30 min. The microorganisms were washed four times as described above and then subjected to elution with 7% SDS for 1 min. Proteins eluted from the 7% SDS buffer were detected by the antiserum to PcLec3.

### Carbohydrate-binding assay

Binding of PcLec3 to carbohydrates was performed using an enzyme-linked immunosorbent assay (ELISA) as described in a previous study^34^. Briefly, 4 μg of LPS, LTA, or PGN was used to coat each well of the microtiter plate. Plates were then blocked with 200 μl of BSA (1 mg/ml) in TBS for 2 h at 37 °C and washed four times with TBS. Recombinant PcLec3 was diluted two-fold with TBS containing 0.1 mg/ml BSA and then added to the microplates (50 μl/well). The plates were incubated at room temperature for 3 h before they were washed four times with TBS. The rabbit anti-Pc-Lec1 antiserum (1:300 dilution with TBS containing 0.1 mg/ml BSA) was added (100 μl/well), and the plates were incubated for 1 h at 37 °C before the wells were washed four times with TBS. The peroxidase-conjugated goat anti-rabbit IgG (2000× dilution) was added to each well, and the plates incubated for 1 h at 37 °C before they were washed as previously described. The color reaction was developed by 3,3′,5,5′-tetramethylbenzidine (Sigma) liquid substrate in citric acid–Na_2_HPO_4_ buffer (0.01%) and then stopped by adding 2 M H_2_SO_4_. The absorbance was read at 450 nm. Control experiments were performed using TBS instead of the recombinant proteins. The assays were performed in triplicate. The same method was used for the binding assay of the recombinant IG and CTLD.

### Hemocyte immunocytochemistry

*V. anguillarum* was injected in to crayfish (2 × 10^7 ^cells/crayfish). Hemolymph was collected at 0, 0.5, and 1 h PI from the ventral sinus using a 1 ml sterile syringe containing 500 μl anticoagulant. Hemocytes were isolated by centrifuging the hemolymph at 800 × *g* for 5 min at 4 °C and then washed two times with the anticoagulant and crayfish-saline (CFS; 0.22 M NaCl, 5.4 mM KCl, 2.6 mM MgCl_2_, 2 mM NaHCO_3_) as described by Jiravanichpaisal^18^. The hemocytes were dropped onto the glass slides and fixed immediately with 4% paraformaldehyde in PBS (140 mM NaCl; 2.7 mM KCl; 10 mM Na_2_HPO4; 1.8 mM KH_2_PO_4_, pH 8.0) for 30 min at room temperature. After washing with PBS, hemocytes were blocked with 1% BSA in PBS and then incubated with polyclonal rabbit antiserum against PcLec3 (1:100 dilution) overnight at 4 °C. The antibody binding of the fluorescein isothiocyanate (FITC)-conjugated goat anti-rabbit IgG (1:10000 in PBS) was visualized by fluorescence microscope. The hemocyte nuclei were incubated in DAPI (4′, 6-diamidino-2-phenylindole; 1:10000 in PBS) for 10 min for counterstaining.

### Detection of cell binding and adhesion

The recombinant PcLec3, IG, and CTLD were dialyzed in CFS and then separately injected into the abdominal segment of crayfish (100 μg/crayfish). V19, a recombinant protein with a His-tag was used as the control. Hemolymph was extracted from the crayfish at 15 min, 30 min, and 1 h PI, and the hemocytes were used to perform the immunohistochemistry as described above. The anti-His-tag antibody was used to detect the recombinant proteins, while the FITC-conjugated goat anti-mouse IgG was used as the secondary antibody.

### Phagocytosis assay *in vivo*

*V. anguillarum* was incubated with 4% paraformaldehyde for 5 h at 25 °C and then stained using DAPI. The phagocytosis of crayfish hemocytes was assayed. Briefly, PcLec3 (500 μg) was incubated with prepared *V. anguillarum* suspension (2 × 10^8 ^cells) to allow the proteins to bind to the bacteria. After washing the redundant protein with CFS, the bacteria with rPcLec3 were resuspended in CFS (2 × 10^8 ^cells/ml) and injected into crayfish (50 μl). Hemocytes from the three crayfishes were extracted at 15 min, 30 min, 1 h, and 2 h PI, alternately washed and centrifuged twice, and then resuspended in CFS (10^7 ^cells/ml). Fluorescence microscopy was used to examine the hemocyte phagocytosis. The hemocytes (at least 200 cells) were counted at 600× magnification. The same assay was performed as described above for rIG, rCTLD, and rVP19 to test their cell phagocytosis activity, with rVP19 as the control. At least 200 hemocytes were counted for each group under the microscope. Phagocytic percentage was calculated as the ratio of the number of cells ingesting the bacteria and the number of cells observed, which was multiplied by 100%. The assays were conducted in triplicate.

### RNA interference

A pair of primers (iF and iR), each linked to the T7 promoter, was used to amplify the templates for dsRNA synthesis. The transcription mixture (50 μl) contained 8 μg of the template, 2.4 μl of each NTP (100 mM), 60 U of T7 RNA polymerase, 80 U of the RNase inhibitor, and 20 μl of 5 × transcription buffer. The mixture was incubated at 37 °C for 8 h, and then 8 U of DNase I was added to remove the templates. After phenol/chloroform extraction and ethanol precipitation, the dsRNA was dissolved in RNase-free water. A total of 50 μg of dsRNA was injected into the crayfish, and the injection was repeated after 24 h. Following the last injection, the mRNA or total proteins from hemocytes was isolated, and the RNAi efficiency was determined by RT-PCR or western blot analysis. The dsGFP RNA was used as control.

### Bacteria clearance assay

The bacteria clearance assay was performed according to a previously described method with slight modifications^30^. Briefly, the purified proteins (50 μg each) were used to coat 1 × 10^8 ^ CFU of *V. anguillarum* at 28 °C for 1 h with gentle rotation. After thorough washing, the bacteria were resuspended in 1 ml PBS. This bacteria suspension (100 μl/crayfish) was injected into the abdominal segment of each crayfish (6 g to 7 g). Hemolymph was then collected after 15 min. After serial dilution, hemolymph was plated onto LB agar plates to count the number of bacteria. For the RNAi group, the same amounts of uncoated and coated bacteria were used.

## Additional Information

**How to cite this article**: Zhang, X.-W. *et al*. A C-type lectin with an immunoglobulin-like domain promotes phagocytosis of hemocytes in crayfish *Procambarus clarkii. Sci. Rep.* **6**, 29924; doi: 10.1038/srep29924 (2016).

## Supplementary Material

Supplementary Information

## Figures and Tables

**Table 1:** Sequences of the primers used in this study.

Primer	Sequence (5′–3′)*	Direction	Position (bp)
ExF	TACTCAGAATTCTCTATCATCTTCGACGTCGCC	Forward	61–81
ExR	TACTCACTCGAGTCACATTATCTGACAAATGAA	Reverse	739–759
IGEXF	TACTCAGAATTCGTCGCCGACGTCGAGGTGACG	Forward	76–96
IGEXR	TACTCACTCGAGTCAAGACGCCAGCAGCGTCCG	Reverse	362–379
CTLDEXF	TACTCAGAATTCTGTCCCGATCCGTTCGTGCTG	Forward	410–430
CTLDEXR	TACTCACTCGAGTCACTGACAAATGAAGCTGCC	Reverse	733–750
RealF	CCGTCACTGTTTATGGGAGAA	Forward	141–161
RealR	CCAACCACCCAGTTCCAG	Reverse	400–417
iF	GCGTAATACGACTCACTATAGGTTGCAATTATCTGGGACCA	Forward	512–530
iR	GCGTAATACGACTCACTATAGGCCTCAACTTTGCCTATATG	Reverse	1024–1042
GFPiF	GCGTAATACGACTCACTATAGGTGGTCCCAATTCTCGTGGAAC	Forward	
GFPiR	GCGTAATACGACTCACTATAGGCTTGAAGTTGACCTTGATGCC	Reverse	
18SF	ACCGATTGAATGATTTAGTGAG	Forward	
18SR	TACGGAAACCTTGTTACGAC	Reverse	

^a^The underlined nucleotides indicate the locations of restriction endonucleases.
