# Supplementary material for: A C-type lectin with an immunoglobulin-like domain promotes phagocytosis of hemocytes in crayfish Procambarus clarkii
Source: Sci Rep. 2016 Jul 14;6:29924. doi: 10.1038/srep29924 (PMC4944128; doi:10.1038/srep29924)
Supplement: Supplementary Information [file srep29924-s1.pdf]

A C-type lectin with an immunoglobulin-like domain promotes phagocytosis of hemocytes in crayfish *Procambarus clarkii*

Xiao-Wen Zhang [1,*], Yue Wang[1], Xian-Wei Wang [2], Lei Wang [2], Yi Mu [2], Jin-Xing Wang [2,*]

[1] College of Life Science, Henan Normal University, Xinxiang, Henan, 453007, China

[2] Shandong Provincial Key Laboratory of Animal Cells and Developmental Biology, School of life science, Shandong University, Jinan, Shandong, 250100, China

*Corresponding author
Dr. Xiaowen Zhang
College of Life Sciences
Henan Normal University
Xinxiang, Henan 453007
China
Tel: 86-13949605978
E-mail address: zxwhtu@163.com

Dr. Jin-Xing Wang
School of Life Sciences
Shandong University
Jinan, Shandong 250100
China
Tel/ Fax: 86-531-88364620
E-mail address: jxwang@sdu.edu.cn

Figure S1. (A) The sequences of IG from three mammals (*Monodelphis domestica* AF116930, *Trichosurus vulpecula* AY074424, and *Macropus eugenii* EF599616) and a cell adhesion protein (CA) in *Ixodes scapularis* XM_002399665 were chosen for the alignment of IG in PcLec3 by MEGA 4.0. Asterisks indicate the two cysteine residues that form the IG typical intrachain disulfide bridge and the tryptophan residue packed against the disulfide bond. (B) Different species of C-type lectin sequences were selected from the NCBI BLAST search program. The phylogenetic tree of CTLD with other animal C-type lectins were obtained using MEGA 4.0.

Figure S2. Recombinant expression and purification of PcLec3 as well as its immunoglobulin-like domain (IG) and C-type lectin domain (CTLD. Lane (a), lysate of *Escherichia coli* with pET30a sequence (*PcLec3*, IG, or CTLD) without induction. Lane (b), lysate of *E. coli* with pET30a sequence induced with IPTG (isopropyl β-D-1-thiogalactopyranoside). Lane (c), purified rPcLec3, IG, or CTLD by His-bind resin chromatography. Lane (d), protein molecular weight marker.

Figure S1

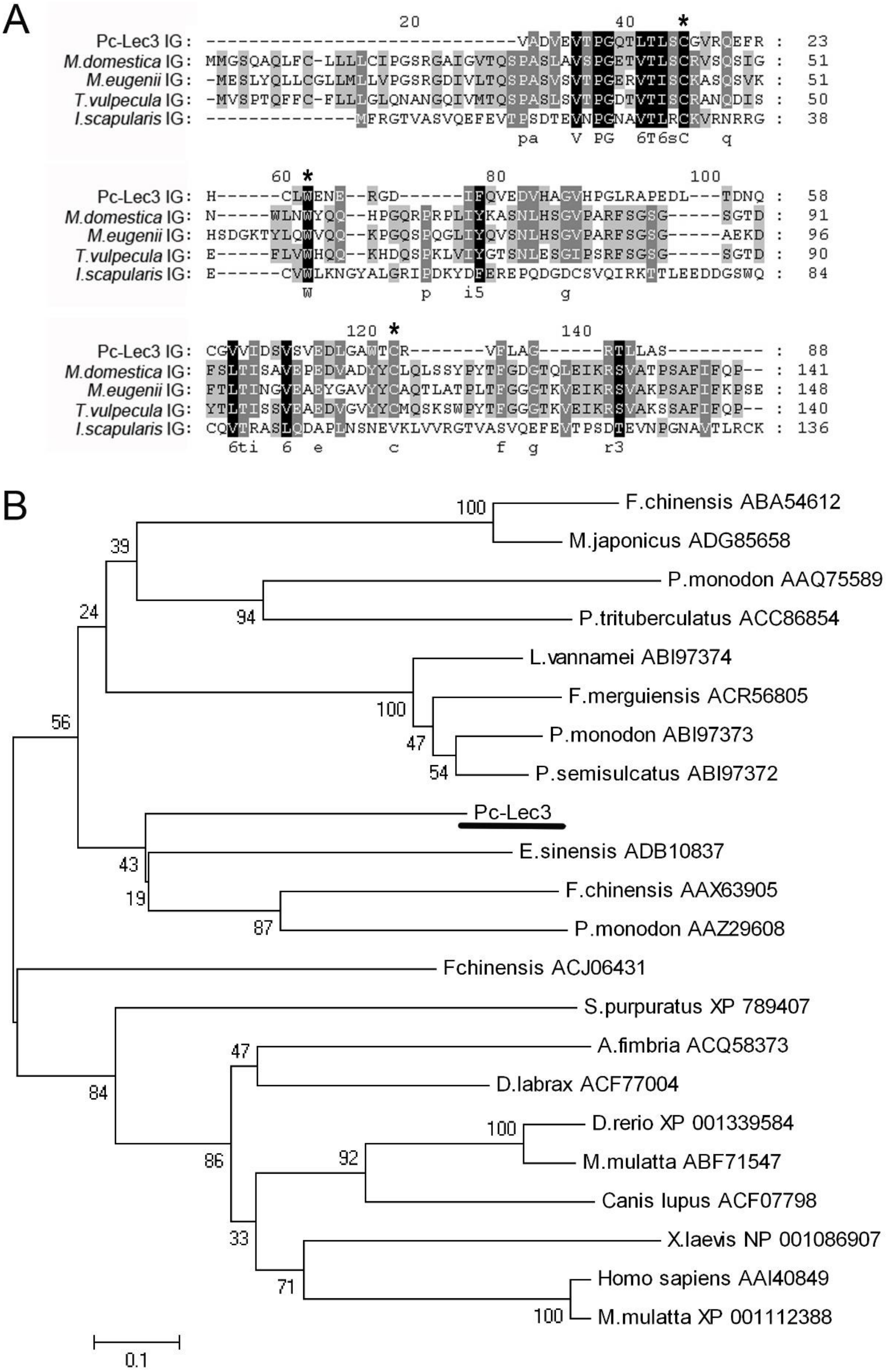
A
20
40
Pc-Lec3 IG : ----------------------------VADVEVTPGQTLTLSCGVRQEFR : 23
M.domestica IG : MMGSQAQLFC-LLLLCIPGSRGAIGVTQSPASLAVSPGETVTLSCRVSQSIG : 51
M.eugenii IG : -MESLYQLLCGLLMLLVPGSRGDIVLTQSPASVSVTPGERVTISCKASQSVK : 51
T.vulpecula IG : -MVSPTQFFC-FLLLGLQNANGQIVMTQSPASLSVTPGDTVTISCRANQDIS : 50
I.scapularis IG : --------------MFRGTVASVQEFEVTPSDTEVNPGNAVTLRCKVRNRRG : 38
pa V PG 6T6sC q
60
80
100
Pc-Lec3 IG : H------CLWENE--RGD------IFQVEDVHAGVHPGLRAPEDL---TDNQ : 58
M.domestica IG : N-----WLNWYQQ--HPGQRERPLIYKASNLHSGVPARFSGSG-----SGTD : 91
M.eugenii IG : HSDGKTYLQWVQQ--KPGQSEQGLIYQVSNLHSGVPARFSGSG-----AEKD : 96
T.vulpecula IG : E-----FLVWHQQ--KHDQSEKLVIYGTSNLESGIPSRFSGSG-----SGTD : 90
I.scapularis IG : E------CVWLKNGYALGRIEDKYDFEREPQDGDCSVQIRKTTLEEDDGSWQ : 84
W p i5 g
120
140
Pc-Lec3 IG : CGVVIDSVSVEDLGAWTCR-------VFLAG------RTLLAS--------- : 88
M.domestica IG : FSLTISAVEPEDVADYYCLQLSSYPYTFGDGTQLEIKRSVATPSAFIFQP-- : 141
M.eugenii IG : FTLTINGVEAEYGAVYYCAQTLATPLTFGGGTKVEIKRSVAKPSAFIFKPSE : 148
T.vulpecula IG : YTLTISSVEAEDVGVYYCMQSKSWPYTFGGGTKVEIKRSVAKSSAFIFQP-- : 140
I.scapularis IG : CQVTRASLQDAPLNSNEVKLVVRGTVASVQEFEVTPSDTEVNPGNAVTLRCK : 136
6ti 6 e c f g r3
B
F.chinensis ABA54612
M.japonicus ADG85658
P.monodon AAQ75589
P.trituberculatus ACC86854
L.vannamei ABI97374
F.merguiensis ACR56805
P.monodon ABI97373
P.semisulcatus ABI97372
Pc-Lec3
E.sinensis ADB10837
F.chinensis AAX63905
P.monodon AAZ29608
Fchinensis ACJ06431
S.purpuratus XP 789407
A.fimbria ACQ58373
D.labrax ACF77004
D.rerio XP 001339584
M.mulatta ABF71547
Canis lupus ACF07798
X.laevis NP 001086907
Homo sapiens AAI40849
M.mulatta XP 001112388
100
39
94
24
100
47
54
56
43
19
87
84
47
100
86
92
33
71
100
0.1

Figure S2

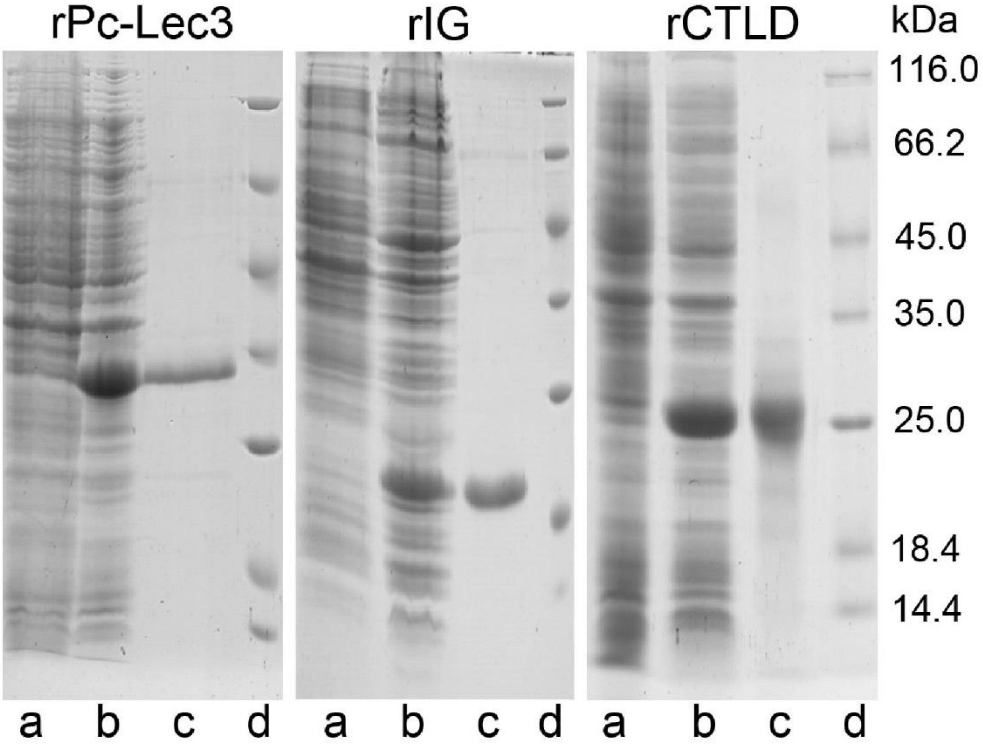
rPc-Lec3
rIG
rCTLD
kDa
116.0
66.2
45.0
35.0
25.0
18.4
14.4
a b c d a b c d a b c d
